# Review of Cases and a Patient Report of Myiasis with Tracheostomy, Peru 

**DOI:** 10.3201/eid2203.151631

**Published:** 2016-03

**Authors:** Virgilio E. Failoc-Rojas, Heber Silva-Díaz

**Affiliations:** Universidad Nacional Pedro Ruiz Gallo, Lambayeque, Peru (V.E. Failoc-Rojas, H. Silva-Díaz);; Hospital Regional Lambayeque, Lambayeque (H. Silva-Díaz)

**Keywords:** myiasis, screw worm infection, ectoparasitic infestations, tracheostomy, developing country, *Cochliomyia hominivorax*, parasites, larva, Peru

**To the Editor:** Myiasis is the infestation in humans of larvae of flies (order Diptera). These larvae can infect skin, necrotic tissues, and natural cavities of living persons. Myiasis can be primary if it infects intact skin or secondary if it infects a previous injury. Depending on the degree of parasitism, myiasis may be obligatory (requiring a live host for parasite survival), facultative (developing in live or dead organic matter), or accidental (developing accidentally in an inappropriate host) (*1*). In South America, the species that most frequently cause myiasis are *Dermatobia hominis* and *Cochliomyia hominivorax*.

Factors contributing to development of myiasis are low socioeconomic status, unhealthy environments, advanced age, alcoholism, neurologic diseases, and lack of personal hygiene (*1**,**2*). Myiasis may occur different tissues, but reports of myiasis of the tracheal stoma are rare. We searched PubMed, MedLine, Lilacs, Scopus, and Google Scholar databases for scientific articles published in English or Spanish languages during 1990–2015 by using the search term “myiasis and tracheostomy.” We found reports of 10 patients (Table).

**Table:** Reports in the literature about myiasis associated with tracheostomy, by date of publication*

**Country**	**Patient age, y/sex**	**Associated conditions**	**Fly species**	**Year of publication**	**Reference†**
**Canada**	85/F	Comatose state for 2 mo	Unidentified	1993	(*3*)
**Italy**	57/M	Persistent vegetative state	*Lucilia caesar*	2006	(*4*)
**Brazil**	49/M	Neck carcinoma	*Cochliomyia hominivorax*	2011	(*5*)
**India**	78/M	Tracheostomy by car accident	*Chrysomya bezziana*	2011	(*6*)
**Argentina**	8/NA	Cerebral palsy	Unidentified	2012	(*7*)
**India**	52/M	Laryngeal cancer	*Musca domestica* (housefly)	2013	(*8*)
**India**	73/NA	Carcinoma supraglottis and diabetes	*Chrysomya bezziana*	2013	(*9*)
**Turkey**	86/F	Poor hygienic condition and tetraplegia	*Lucilia caesar*	2014	(*10*)
**India**	57/M	Proliferative ulcer on vocal cords and glottic stenosis	*Chrysomya bezziana*	2015	(*11*)
**Italy**	5/M	Werdnig-Hoffmann disease	*Sarcophaga argyrostoma*	2015	(*12*)
**Peru**	67/M	Esophageal cancer	*Cochliomyia hominivorax*		This study

*NA, not available. **†Sources: PubMed, Medline, Scopus, LILACS, and Google Scholar. References *11,12* are in the ****Technical Appendix****.**

We also report a case of tracheostomal myiasis in a 67-year-old man from Túcume, Peru. The patient had a history of esophageal tumor lesion with considerable airway stenosis related to upper esophageal cancer (stage III). Six months before onset of myiasis, he had respiratory difficulty caused by obstructed airway and underwent a tracheostomy and gastrostomy. When the patient was admitted to the emergency department of a hospital in Lambayeque, located ≈35 km from the patient’s home, mobile larvae were present at the tracheostomy site, which also contained brown secretions with traces of blood and obvious signs of inflammation. A cervical abscess surrounded by necrotic tissue was visible, which, according to family members, developed after the larval infection. We manually removed the larvae and began treatment with ivermectin orally (1 dose, 200 µg/kg), ceftriaxone orally (2 g/d), and metronidazole intravenously (500 mg every 8 h). Three days after the patient started treatment, the tracheostomy tube was surgically removed for changing, and a large number of dead larvae were then observed and removed. The patient showed no signs of septicemia. He had slight relative eosinophilia (6%), but his hemoglobin and leukocyte levels were within reference ranges; the larvae would be unable to penetrate cells at these levels. The patient improved with no clinical symptoms of cervical abscess or evidence of phlogosis. He was discharged 9 days after admission, with a postdischarge treatment of oral metronidazole (500 mg every 12 h for 3 d). 

Three specimens of larvae were sent to the hospital’s parasitology laboratory, which identified the larvae as *C. hominivorax* stage L-3 (infection began with fly oviposition ≈6 days before admission; L-1, L-2, and L-3 are stages of larval development from hatching until pupation, requiring ≈7 days). The larvae were 10 mm × 3 mm and had a cylindrical, pale yellow body segmented with pigmented tracheal trunks visible in the last 4 posterior segments. Microscopic examination showed that the anterior end had a prominent jaw and segments with small bands of cuticular spines; the rear end had exposed spiracles, each with 3 straight grooves and open peritrematic membranes (reference *13* in the Technical Appendix).

The life cycle of *C. hominivorax* is similar to any other species in the Diptera order. Open wounds and body orifices (e.g., a tracheostomy) emitting odors from natural secretions are conducive for oviposition by flies and development of myiasis. A study from Brazil mentions that open wounds are the leading cause of development of the *C. hominivorax* parasite (*2*). Chronic extensive wounds are often infested by *C. hominivorax* (*2**,**5*). 

Myiasis infection is concerning because it can lead to secondary infections such as *Escherichia coli*, *Serratia marcescens*, and *Enterococcus faecalis* (*6*). The infection is most dangerous when patients have concurrent conditions such as immunosuppression.

Treatment of myiasis involves manual removal of larvae and surgical debridement, in conjunction with ivermectin and systemic broad-spectrum antimicrobial drugs to prevent secondary infections (*1**,**2*). Treatment with ivermectin can kill the larvae (*1*; references *14,15* in the Technical Appendix) and result in considerable reduction of larvae in infested wounds. Ivermectin has a broad antiparasitic spectrum that causes immobilization of parasites by inducing tonic paralysis of the parasite’s muscles, mainly at the pharyngeal level, resulting in the death of the parasites by suffocation and starvation. 

For the patient in this report, the single oral dose (0.2 mg/kg) of ivermectin was an effective treatment for myiasis. However, to control the underlying disease and prevent recurrences, ivermectin should be used with oral antimicrobial drugs and wound care when the wound has a high number of larvae, which are associated with bacterial infections (*4**,**5*).

For bedridden patients, patients with superficial wounds who live in myiasis-endemic areas, or patients who undergo a tracheostomy or have open wounds, health workers and caregivers should consider preventive care of wounds, which are risk factors for myiasis infection. This care consists of suitable wound dressing and proper personal and environmental hygiene.

Technical Appendix Additional References
