# Supplementary material for: Review of Cases and a Patient Report of Myiasis with Tracheostomy, Peru
Source: Emerg Infect Dis. 2016 Mar;22(3):563–5. doi: 10.3201/eid2203.151631 (PMC4766876; doi:10.3201/eid2203.151631)
Supplement: Technical Appendix — Additional References [file 15-1631-Techapp-s1.pdf]

Article DOI: http://dx.doi.org/10.3201/eid2203.151631

Review of Cases and a Patient Report of Myiasis with Tracheostomy, Peru

Technical Appendix

Additional References

11. Manickam A, Sengupta S, Saha J, Basu SK, Das JR, Sannigrahi R. Myiasis of the tracheostomy wound: a case report with review of literature. Otolaryngology. 2015;5:2. http://dx.doi.org/10.4172/2161-119X.1000198

12. Severini F, Nocita E, Tosini F. Myiasis of the tracheostomy wound caused by Sarcophaga (Liopygia) argyrostoma (Diptera: Sarcophagidae): molecular identification based on the mitochondrial cytochrome c oxidase I gene. J Med Entomol. 2015;52:1357–60. PubMed http://dx.doi.org/10.1093/jme/tjv108

13. Mathison BA, Pritt BS. Laboratory identification of arthropod ectoparasites. Clin Microbiol Rev. 2014;27:48–67. PubMed http://dx.doi.org/10.1128/CMR.00008-13

14. Dourmishev AL, Dourmishev LA, Schwartz RA. Ivermectin: pharmacology and application in dermatology. Int J Dermatol. 2005;44:981–8. PubMed http://dx.doi.org/10.1111/j.1365-4632.2004.02253.x

15. Duque FL, Ardila CM. Oral myiasis caused by the screwworm *Cochliomyia hominivorax* treated with subcutaneous ivermectin and creolin: report of six cases after trauma. Dent Traumatol. 2011;27:404–7. PubMed http://dx.doi.org/10.1111/j.1600-9657.2011.01004.x
